# Functional TRAIL receptors in monocytes and tumor-associated macrophages: A possible targeting pathway in the tumor microenvironment

**DOI:** 10.18632/oncotarget.9340

**Published:** 2016-05-13

**Authors:** Manuela Liguori, Chiara Buracchi, Fabio Pasqualini, Francesca Bergomas, Samantha Pesce, Marina Sironi, Fabio Grizzi, Alberto Mantovani, Cristina Belgiovine, Paola Allavena

**Affiliations:** ^1^ Department of Immunology and Inflammation, IRCCS-Humanitas Clinical and Research Center, 20089 Rozzano, Milano, Italy; ^2^ Humanitas University, 20089 Rozzano, Milano, Italy

**Keywords:** TRAIL, TRAIL receptors, apoptosis, tumor-associated macrophages, targeting macrophages

## Abstract

Despite the accepted dogma that TRAIL kills only tumor cells and spares normal ones, we show in this study that mononuclear phagocytes are susceptible to recombinant TRAIL via caspase-dependent apoptosis. Human resting monocytes and *in vitro*-differentiated macrophages expressed substantial levels of the functional TRAIL receptors (TRAIL-R1 and TRAIL-R2), while neutrophils and lymphocytes mostly expressed the non-signaling decoy receptor (TRAIL-R3). Accordingly, exclusively monocytes and macrophages activated caspase-8 and underwent apoptosis upon recombinant TRAIL treatment. TRAIL-Rs were up-regulated by anti-inflammatory agents (IL-10, glucocorticoids) and by natural compounds (Apigenin, Quercetin, Palmitate) and their treatment resulted in increased TRAIL-induced apoptosis. In mice, the only signaling TRAIL-R (DR5) was preferentially expressed by blood monocytes rather than neutrophils or lymphocytes. In both mice and humans, Tumor-Associated Macrophages (TAM) expressed functional TRAIL-R, while resident macrophages in normal tissues did not. As a proof of principle, we treated mice bearing a murine TRAIL-resistant fibrosarcoma with recombinant TRAIL. We observed significant decrease of circulating monocytes and infiltrating TAM, as well as reduced tumor growth and lower metastasis formation. Overall, these findings demonstrate that human and murine monocytes/macrophages are, among leukocytes, uniquely susceptible to TRAIL-mediated killing. This differential susceptibility to TRAIL could be exploited to selectively target macrophages in tumors.

## INTRODUCTION

In the last decade the inflammatory tumor microenviroment has been recognized as a hallmark of cancer and many efforts concentrated in counteracting or limiting this cancer-related inflammation [1–4]. Several studies have now established that local Tumor Associated Macrophages (TAM) fuel the build up of the inflammatory milieu and favour, rather than inhibit, disease progression [5, 6]. In fact, TAM density in human tumors is usually associated with poor patient prognosis and resistance to therapies [1, 3, 7, 8]. Targeting of TAM for therapeutic purposes has been extensively pursued and is providing positive results in various experimental settings, either as monotherapy or combined with conventional and anti-angiogenic therapies. A number of different approaches has been used to target TAM in tumors: inhibition of their recruitment by using inhibitors of CCL2, alone or in combination therapy [9, 10]; the use of anti-CSF1R monoclonal antibodies or antagonists to the CSF1R tyrosine kinase [11–13]; as well as strategies aimed to their direct elimination, by inducing apoptosis or inhibition of vital pathways [14, 15], including inhibition of the mTOR pathway [16–18]. Finally a different approach is the re-programming of their functional phenotype to re-direct TAM towards cytotoxic effectors [19, 20]. Our group recently reported that trabectedin, a registered anti-tumor agent of marine origin, has interesting peculiar effects on the tumor micro-environment, in addition to block cell cycles in proliferating tumor cells. *In vivo* studies in mice and evidence in human patients revealed that at least part of its anti-tumor activity is mediated through the reduction of macrophages in tumors. Mechanistically, trabectedin selectively induced apoptosis in monocytes and macrophages via activation of caspase-8 [14]. Caspase-8 is the prime molecule involved in the extrinsic apoptotic pathway which initiates downstream of death receptors expressed on cell membranes.

Apoptosis activation is a tightly regulated process which involves a number of distinct functional receptors, some non-signaling or decoy receptors, in addition to several adaptor or regulatory proteins. In the case of the extrinsic apoptosis, two major classes of membrane-associated death receptors have been identified: Fas, the receptor for the Fas ligand, and various receptors for the TNF-related apoptosis-inducing ligand (TRAIL). Among the latter, there are two functional receptors, TRAIL R1 (DR4) and TRAIL R2 (DR5) having a death domain in their intracellular portion, and three non-signaling receptors, TRAIL R3 (Dcr1), TRAIL R4 (Dcr2) and osteoprotegerin (OPG), which lack the death domain and are unable to induce apoptosis, but compete with functional receptors for TRAIL binding [21–23]. Binding of TRAIL causes functional receptor oligomerization, with formation of DISC (Death Inducing Signalling Complex) and consequent activation of a caspase cascade that eventually leads to apoptotic cell death [21].

The current dogma is that TRAIL kills tumor cells *in vitro* and *in vivo* but spares normal cells that are insensitive to its apoptotic effect [24–28]. Our finding that the compound trabectedin was able to activate caspase-8 and apoptosis selectively in monocytes, contradicted this dogma and raised the issue of a differential death receptor expression in distinct immune cell subsets. Some recent studies have shown that under conditions of bacterial or viral infections, immune cells become susceptible to TRAIL, as observed with HIV-infected T cells and alveolar macrophages during lung infection with Streptococcus pneumonia [29–31]. However, the vulnerability of primary leukocytes under normal homeostatic conditions is largely understudied. This prompted us to perform an in-depth analysis of death receptor expression and modulation in different leukocyte subsets with a special focus on mononuclear phagocytes in the tumor context.

Here we demonstrate that resting monocytes and macrophages differentially express signalling and decoy TRAIL-Rs and are susceptible to TRAIL-induced apoptosis. As a proof-of-principle, tumor-bearing mice treated with recombinant TRAIL had slowed tumor growth and reduced number of TAM in tumors.

## RESULTS

### Characterization of death receptors in human leukocyte subsets

Our initial observation that monocytes can be targeted by the anti-tumor agent trabectedin through extrinsic apoptosis [14] prompted us to define the expression and modulation of death receptors in human and mouse leucocyte subsets.

**Figure 1:**
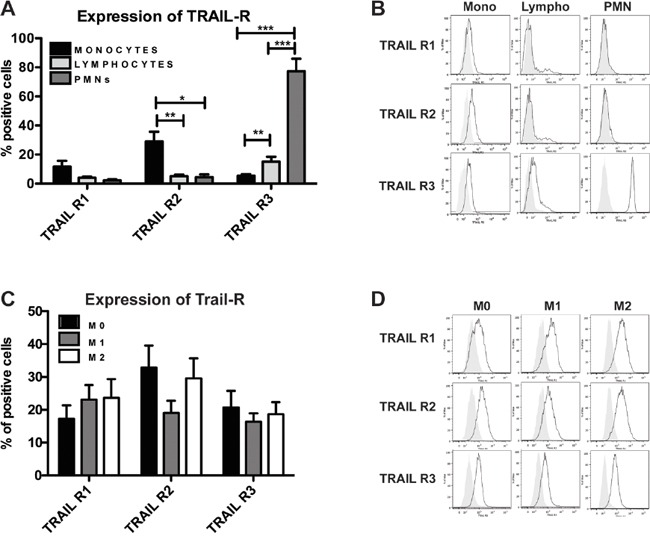
Human monocytes and macrophages express functional TRAIL receptors Flow cytometry analysis of TRAIL receptor (TRAIL-R) expression. **A-B.** Freshly isolated purified monocytes, lymphocytes and granulocytes (PMN); **C-D.** *In vitro* MCSF-differentiated macrophages (M0) and polarized M1 (LPS, IFNγ) and M2 (IL-4) macrophages. In A and C, results are shown as % of positive cells (mean ± SE of 10 experiments). In B and D Representative plots are shown. Statistical analysis: *P < 0.05, ** P < 0.01, *** P < 0.001 (Student's t test).

Freshly isolated purified human blood leukocytes were tested in flow cytometry; the Fas receptor was expressed at high levels in all leucocyte types (Figure S1A), while the expression of TRAIL-Rs was heterogeneous: the functional TRAIL-Rs (TRAIL-R1 and TRAIL-R2) were mainly expressed on monocytes whereas the decoy receptor (TRAIL-R3) was highly expressed on neutrophils and to a lesser extent on T lymphocytes (Figure 1A-1B); of note, in lymphocytes activated with ionomycin and PMA, TRAIL-R3 was greatly increased (Figure S1B). Despite considerable heterogeneity among the donors, the results clearly indicated that the ratio between functional and decoy receptors, the key point determining TRAIL susceptibility, was in favor of functional TRAIL-Rs for monocytes and of the decoy receptor for neutrophils and lymphocytes. The other non-functional TRAIL-Rs (OPG and TRAIL-R4) were not significantly expressed in resting leukocytes (data not shown).

We next investigated TRAIL-Rs in monocyte subsets on the basis of the expression of CD14, MHC II and the chemokine receptor CX3CR1. TRAIL-R2 was similarly expressed among all monocyte subsets, while TRAIL-R1 was higher in CD14^bright^ monocytes, with the exception of CD14^bright^/MHC II^dim^ cells. TRAIL-R3, which is usually very low, was higher in CD14^bright^/CX3CR1^+^ cells (Figure S1C).

In monocyte-derived macrophages, expression of all TRAIL-Rs was up-regulated compared to resting monocytes (Figure 1C-1D); of note, M1 and M2 polarized macrophages had similar expression levels, although M2 cells had higher TRAIL-R2 compared to M1 macrophages.

In conclusion, functional TRAIL-Rs are mostly expressed on monocytes and macrophages, while the decoy R3 is preferentially expressed in neutrophils and lymphocytes.

### TRAIL receptors in human resident tissue macrophages and tumor-associated macrophages

**Figure 2:**
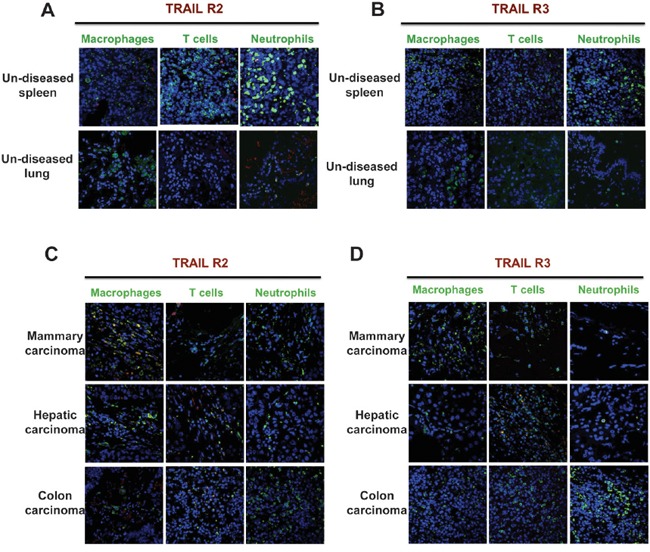
Human tumor-associated macrophages express TRAIL-R2 Immunofluorescence of TRAIL-R2 and TRAIL-R3 and leukocyte markers. **A-B.** Sections of un-diseased human spleen and lung; **C-D.** Sections of human mammary, hepatic and colon carcinoma. Macrophages (CD68), T cells (CD3) and granulocytes (myeloperoxidase) are marked in green, TRAIL-R2 and R3 in red and nuclei in blue. Macrophages from tumor tissues, but not from normal tissues, express TRAIL-R2 and do not express TRAIL-R3.

We next investigated death receptor expression in a series of normal and tumor tissues, by immunofluorescence. In normal human spleen and lungs, TRAIL-R2 and TRAIL-R3 were barely detectable (Figure 2A-2B). A similar pattern was observed for TRAIL-R1 (not shown). In human tumor tissues, TRAIL-R2 was expressed in the majority of macrophages in hepatic and mammary carcinoma samples, and less frequently in colon carcinoma (Figure 2C), but it was not expressed by infiltrating lymphocytes and neutrophils. TRAIL-R3 in tumor tissues was not expressed by any leukocyte type, with the exception of T lymphocytes in hepatic carcinoma (Figure 2D). Altogether these data show that tissue resident macrophages have no or low expression of functional TRAIL-Rs, while TAM within tumor tissues, but not other leukocytes, do express mainly TRAIL-R2.

### TRAIL receptors are modulated by anti-inflammatory stimuli in human monocytes/macrophages

**Figure 3:**
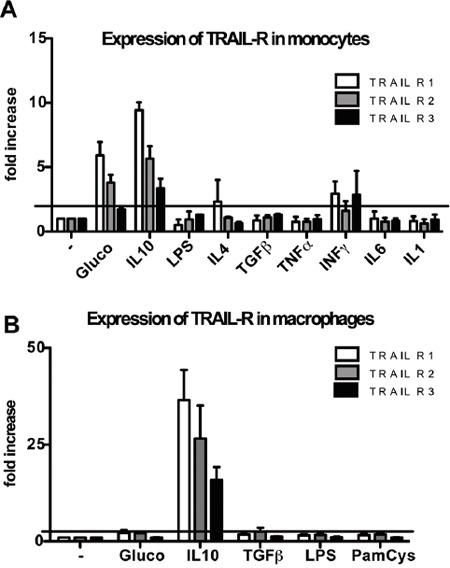
Modulation of TRAIL receptors in human monocytes and macrophages Glucocorticoids (10^−7^M, 24 hrs) induce up-regulation of TRAIL-R1-2 in monocytes while IL-10 (25 ng/ml, 24 hrs) induces up-regulation of TRAIL-R1-2 and to a lesser extent of TRAIL-R3 in monocytes (A) and macrophages (B). Results are shown as fold increase relative to untreated cells (mean ± SE of 3 different experiments). Statistical analysis: *P < 0.05, ** P < 0.01, *** P < 0.001 (Student's t test).

TRAIL receptor modulation was tested upon treatment *in vitro* of purified monocytes with several cytokines and prototypic stimuli. We observed that LPS, IL-1, TNFα, IL-6, as well as IL-4 and TGFβ did not modulate TRAIL-R1-2 or the decoy R3, which were actually slightly down-modulated. In contrast, IL-10 and glucocorticoids, strongly up-regulated TRAIL-R1-2 and, to a lesser extent, TRAIL-R3. IFNγ had a mild inducing effect on TRAIL-R1 and R3. The results are shown in Figure 3A and are expressed as fold increase over untreated cells.

In macrophages differentiated *in vitro*, IL-10, but not glucocorticoids, induced TRAIL-R up-regulation, while LPS, Pam-Cys and TGFβ had no effect (Figure 3B). Overall, these data indicate that TRAIL-Rs are positively modulated by anti-inflammatory mediators.

### Monocytes and macrophages are susceptible to TRAIL treatment

**Figure 4:**
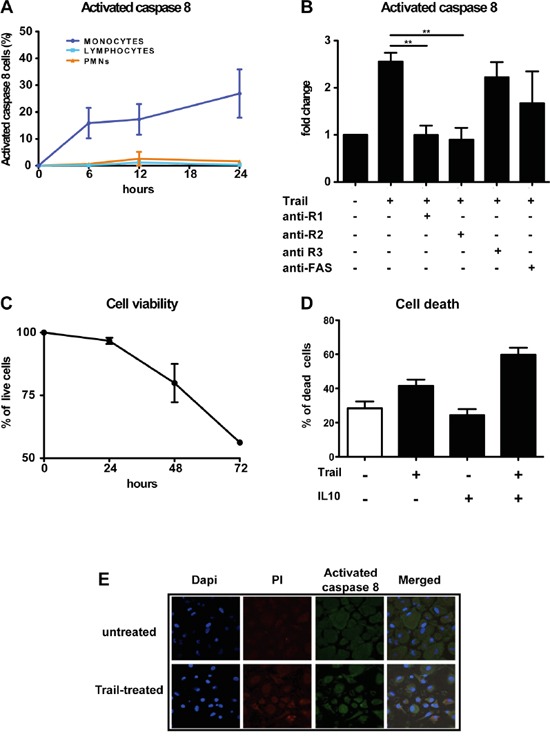
rhTRAIL induces caspase-8 activation and apoptosis selectively in human monocytes and macrophages **A.** Activation of caspase-8 analyzed by flow cytometry after rhTRAIL (SuperKiller) treatment (300 ng/ml); monocytes but not granulocytes and lymphocytes activate caspase-8. Results are shown as % of positive cells (mean ± SE of 5 different donors for monocytes and lymphocytes, 2 for granulocytes). **B.** Activation of caspase-8 in monocytes analyzed by flow cytometry; activation is blocked by anti-TRAIL-R1/R2 antibodies. Results are shown as fold change relative to untreated cells. **C.** Cell viability of monocytes treated with rhTRAIL for up to 72 hrs evaluated with Annexin/PI staining by flow cytometry; results are shown as % of live cells (Mean ± SE of 4 experiments). **D.** Monocyte death upon 24 hrs treatment with rhTRAIL (300 ng/ml) and IL-10 (25 ng/ml) alone or in combination; results are shown as % of dead cells (Mean ± SE of 3 experiments). Statistical analysis: *P < 0.05, ** P < 0.01, *** P < 0.001 (Student's t test). **E.** Immunofluorescence analysis of Caspase-8 activation in *in vitro* differentiated macrophages after rhTRAIL treatment (300 ng/ml, 12 hrs); Images were taken using confocal microscope: nuclei are shown in blue, PI in red and cleaved-caspase 8 in green.

The extrinsic apoptotic pathway starts with the activation of caspase-8, induced when a death ligand, such as FasL, TNF or TRAIL, binds to its receptors. To understand whether the differential expression of TRAIL-Rs in leucocyte subsets corresponds to a different TRAIL susceptibility, we monitored caspase-8 activation in recombinant TRAIL-treated leukocytes by flow cytometry. We observed that lymphocytes and neutrophils were unable to activate caspase-8 when treated with TRAIL, while monocytes rapidly activated caspase-8 in a time dependent manner (Figure 4A). The involvement of functional TRAIL-Rs was confirmed by using anti-TRAIL-R1-2 blocking antibodies that inhibited caspase-8 activation, while anti-TRAIL-R3 or anti-Fas, used as negative controls, had no effect (Figure 4B). Activation of caspase-8 per se is not sufficient to induce apoptosis; we therefore analyzed the viability of TRAIL-treated monocytes over time. TRAIL induced monocyte apoptotic death in a time dependent manner, reducing their survival by 50% after 72h of treatment (Figure 4C). Furthermore, pre-treatment with IL-10 substantially sensitized monocytes to TRAIL-mediated death (Figure 4D).

Caspase-8 activation and mortality was induced also in cultured macrophages upon TRAIL treatment, as shown by immunofluorescence, but with less efficiency compared to monocytes (Figure 4E); this is probably due to their increased expression of TRAIL-R3 (Figure 1C) and intrinsic resistance to death due to their activated state.

### Modulation of TRAIL receptors and apoptosis by natural compounds

**Figure 5:**
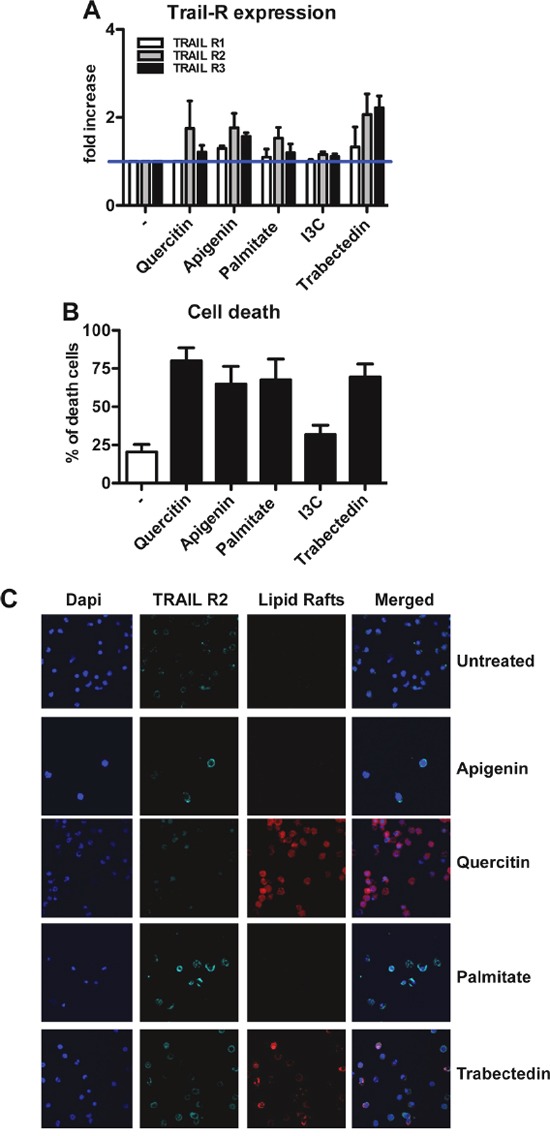
Modulation of TRAIL receptors in human monocytes by natural compounds **A.** Up-regulation of TRAIL-R expression upon treatment with Quercitin, Apigenin, Palmitate and Trabectedin for 24 hrs; results are shown as fold increase relative to untreated monocytes (mean±SE of 5 experiments). Indole-3-carbinole (I3C) has no effect on TRAIL-R modulation. **B.** Quercitin, Apigenin, Palmitate and Trabectedin induce cell death after 12hrs treatment; results are shown as % of dead cells (mean ± SE of 5 experiments). **C.** Immunofluorescence analysis of TRAIL-R2 (light blue) and lipid rafts (red) in treated monocytes. Quercitin and Trabectedin induce TRAIL-R2 co-localization into lipid rafts. Statistical analysis: *P < 0.05, ** P < 0.01, *** P < 0.001 (Student's t test).

It is known that some natural compounds, such as Palmitate and snail venom, are able to activate caspase-8 in a TRAIL-independent manner, through up-regulation and/or aggregation of death receptors [32, 33]. We investigated the effects of Apigenin, Palmitate, Quercitin, Indole-3-Carbinole (I3C) and the anti-tumor agent trabectedin on purified human monocytes. These compounds, with the exception of I3C, indeed induced up-regulation of TRAIL-Rs, in particular TRAIL-R2 (Figure 5A). Higher expression of TRAIL-Rs was paralleled by increased cell mortality (Figure 5B).

To analyze the mechanism of ligand-independent death, we further investigated by immunofluorescence the expression of TRAIL-R2 and its localization on cell membranes. Upon treatment with Quercitin and trabectedin, the increased expression of TRAIL-R2 correlated with its aggregation into lipid rafts (Figure 5C), in contrast Apigenin and Palmitate were not able to induce this aggregation and the increased death likely occurred via increased expression of receptors.

### Characterization of TRAIL receptors in murine leucocyte subsets

**Figure 6:**
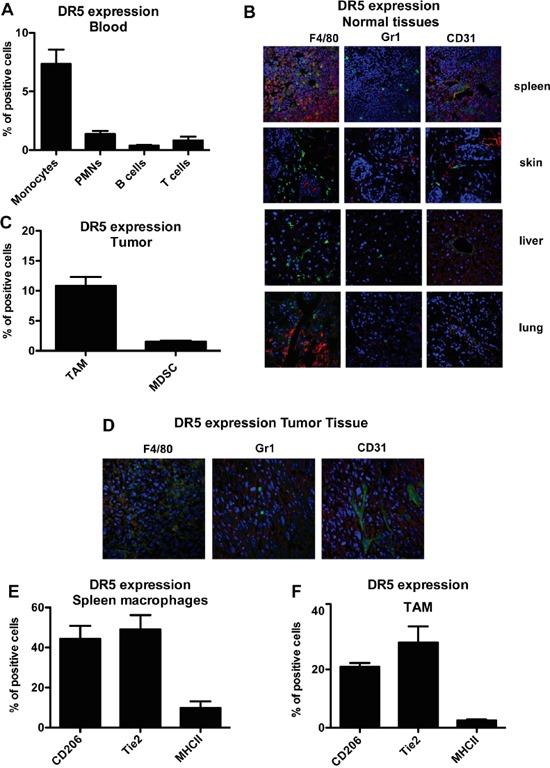
Murine monocytes and macrophages express the functional TRAIL receptor DR5 **A.** Flow cytometry analysis of murine TRAIL-R (DR5) in blood monocytes, granulocytes, T and B lymphocytes; results are shown as % of positive cells relative to total CD45^+^ cells (mean±SE of 29 blood samples). **B.** Immunofluorescence analysis of DR5, macrophages (F4/80), granulocytes (Ly6G) and endothelial cells (CD31) in normal murine tissues, (DR5 in red, F4-80/Ly6G/CD31 in green and nuclei in blu). **C.** DR5 expression in TAM and in MDSC from tumor tissues; results are shown as % of positive cells (Mean ± SD of 3 experiments); **D.** immunofluorescence analysis of DR5 in fibrosarcoma tumor tissues (DR5 in red, F4-80/Ly6G/CD31 in green and nuclei in blu). **E-F.** DR5 expression in CD206+, Tie2^+^, MHCII+ macrophages from spleen (E) and tumor (F); results are shown as % of positive cells (Mean ± SD of 3 experiments). Statistical analysis: *P < 0.05, ** P < 0.01, *** P < 0.001 (Student's t test).

In mice only one functional TRAIL-R is present, DR5 [34]. We first characterized DR5 expression in murine blood leukocyte subsets. As observed in human cells, only murine monocytes expressed appreciable levels of DR5, while the receptor was virtually absent in neutrophils, T and B cells (Figure 6A).

The analysis was performed in two mouse strains (C57BL/6 and DBA2J), with identical results (not shown). Furthermore, *in vitro* treatment of monocytes isolated from bone marrow with recombinant TRAIL decreased their viability (Figure S2A). The apoptotic effect was modest, compared to human monocytes, but consistently observed.

By immunofluorescence of normal tissues (spleen, skin, liver, lung) we observed that DR5 was not or rarely expressed by leukocytes, with the exception of the spleen, where it was present on F4/80^+^ macrophages as well as by some blood vessels, but not on Gr1^+^ myeloid cells (Figure 6B). This finding was confirmed also on disaggregated splenocytes by flow cytometry (Figure S2B).

We next checked DR5 expression leukocytes infiltrating the transplantable murine fibrosarcoma (MN/MCA1). TAM expressed substantial levels of DR5, but not other myeloid cells or vessels, as shown in Figure 6C (flow cytometry on disaggregated cells) and in Figure 6D (immunofluorescence on tumor sections). As we observed that not all F4/80^+^ macrophages were DR5^+^, we investigated other macrophage markers. In the spleen of tumor-bearing mice (Figure 6E) and in tumor tissues (Figure 6F), DR5 was predominantly expressed by CD206^+^ and Tie2^+^ macrophages but not by F4/80^+^ MHCII^+^ cells.

Overall, these data indicate that, also in mice, the functional TRAIL-R DR5 is mainly expressed by monocytes and macrophages, including TAM.

### Targeting of tumor macrophages *in vivo* by recombinant TRAIL

**Figure 7:**
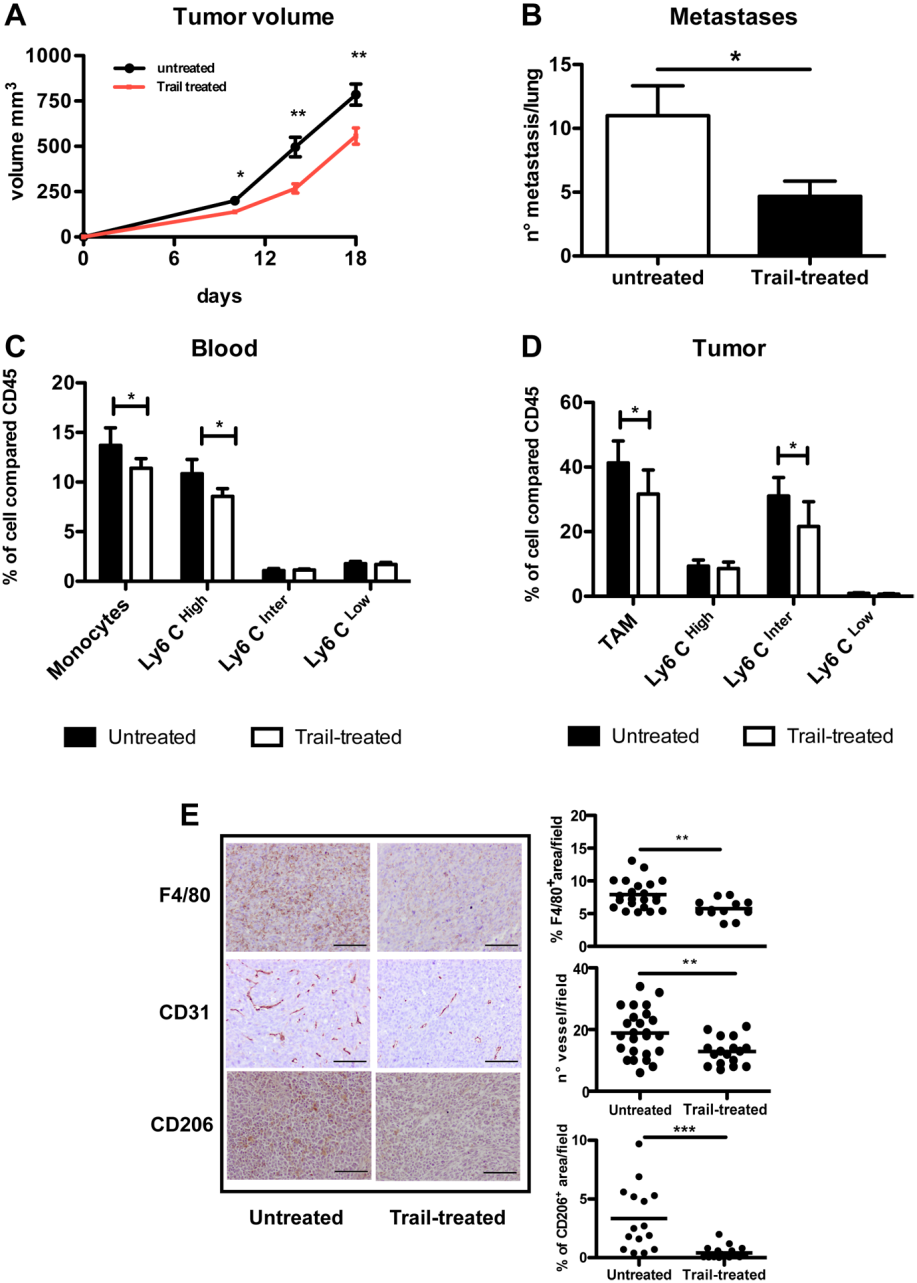
*In vivo* effects of TRAIL treatment on TAM and tumor growth **A-B.** Anti-tumor effect of TRAIL treatment (0,01 g/kg) on primary tumor growth and spontaneous lung metastases (mean ±SE, 7 mice per group; representative experiment of two performed with similar results. **C.** Flow cytometry analysis of blood monocytes from TRAIL-treated or untreated mice; monocytes and in particular Ly6C^High^ expressing monocytes are significantly reduced after 48 hrs treatment. **D.** Flow cytometry analysis of TAM from TRAIL-treated or untreated mice; TAM and in particular the Ly6C^Inter^ subset are significantly reduced after treatment; in both C and D results are shown as % of positive cells relative to total CD45^+^ cells (mean ±SE, 7 mice per group) **E.** Immunohistochemistry of tumor sections from TRAIL-treated and untreated mice stained for F4/80, CD31 and CD206. The immunoreactive area for macrophages (F4/80, CD206) and number of vessels (CD31) are shown on the right panels. Results are calculated as mean from five microscope fields for each sample, 7 mice per group. Images were analyzed using Image-Pro Analyzer software. Original magnification 20X. Statistical analysis: *P < 0.05, ** P < 0.01, *** P < 0.001 (Student's t test).

In order to understand whether the TRAIL-R pathway could be used to target TAM in the tumor micro-environment, we treated mice transplanted with the MN/MCA1 fibrosarcoma with recombinant TRAIL. We previously reported that this murine fibrosarcoma is highly infiltrated by macrophages, which have a significant accelerating effect on tumor progression [14, 35]. We first checked the susceptibility of fibrosarcoma cells to TRAIL treatment *in vitro* and found that they were intrinsically resistant, while the anti-tumor agent trabectedin, used as positive death inducer, considerably reduced cell viability (Figure S3A). TRAIL treatment of mice significantly impaired tumor growth and spontaneous lung metastasis formation (Figure 7A). Resistance of tumor cells to TRAIL, after *in vivo* growth and treatment, was confirmed also *ex vivo* on cultured cells (Figure S3B). In TRAIL-treated mice, the number of blood monocytes, in particular the Ly6C^high^ subset, was significantly decreased 48 hours post-treatment (Figure 7C), as well as the number of TAM in tumors (especially the Ly6C^int^ subset) as detected by flow cytometry (Figure 7D) and by immunohistochemistry (Figure 7E). Figure 7E also shows the marked reduction of CD206^+^ TAM, the most expressing DR5 subsets; furthermore, the vessel network in treated tumors was appreciably reduced.

Taken together these data demonstrate, in a context where tumor cells are not susceptible, that treatment with TRAIL targets *in vivo* monocytes/macrophages and significantly reduces tumor progression.

## DISCUSSION

In this study we performed a comprehensive analysis of TRAIL receptor expression and function in blood leukocyte subsets and in tissue resident and tumor-associated macrophages, in mice and humans.

In blood leukocytes, functional TRAIL-Rs (TRAIL-R1-2) are exclusively expressed by monocytes while neutrophils and T cells have only the decoy receptor TRAIL-R3. The biological significance of this clear-cut segregation is unclear. We can speculate that it is reasonable that monocytes must be kept under strict control, being potentially very reactive cells and with a relatively long life span. Neutrophils, instead, have a life span of few hours in the circulation and the decoy TRAIL-R3 could be helpful to maintain their integrity. Finally, T lymphocytes bear memory for antigens and must be protected from death. Of note, the other death receptor: Fas, is equally expressed and at high levels on all leukocyte subsets. Thus, while functional TRAIL-Rs are present only on monocytes, Fas is present also on neutrophils and T lymphocytes. The regulation of cell susceptibility to Fas ligand-induced apoptosis could be at the ligand rather than at the receptor level, as it appears to be for TRAIL; in fact FasL is produced and regulated in a strict manner, while TRAIL ligand is usually constitutively expressed by several tissues [24, 25, 27, 28], although it can be induced in immune cells upon stimulation with LPS, IFNβ or IFNγ [36].

Death pathways impact importantly on the immune system development and regulation. TRAIL-deficient mice have been reported to have defects in the thymic negative selection and control of central tolerance, with consequences for the development of autoimmune diseases [37, 38] although not confirmed in other studies [39]. Sedger et al. reported that control of aberrant lymphocyte expansion requires both FasL and TRAIL and only the double KO mice show a marked lymphoproliferative disease and severe autoimmunity [40]. Thus TRAIL in tandem with FasL are important to control lymphocyte homeostasis and to limit autoimmunity. As far as innate immunity, and in line with our data on monocytes/macrophages, TRAIL-R KO mice were reported to have boosted innate immune responses with increased levels of IL-12, IFN-α, and IFN-γ after pathogen infection [41].

The ligand TRAIL has been reported to mediate also non-apoptotic effects [42]. Besides the activation of death receptors and downstream caspase-dependent apoptosis, TRAIL can activate pro-survival pathways including NF-kB, PI3K, MAPK [43, 44]. Furthermore, TRAIL-Rs have been implicated in liver-associated macrophage inflammation leading to obesity and insulin resistance [45]. On the other hand, other studies suggested that TRAIL can negatively regulate the inflammatory response [46–48]. In this study we investigated whether TRAIL could modify the constitutive or LPS-induced production of several inflammatory mediators (e.g. IL-1β, IL-6, CXCL8) in human monocytes, but we did not observe any significant modulation (data not shown).

TRAIL-Rs on leukocytes can be up-regulated under infectious conditions, such as in T cells infected by the HIV virus and alveolar macrophages infected by Streptococcus pneumonia [29, 31]. In our study, the analysis of TRAIL-R modulation on monocytes and macrophages revealed that pro-inflammatory cytokines have no significant role, while cytokines with anti-inflammatory properties such as IL-10 and glucocorticoids strongly up-regulate TRAIL-Rs. A possible scenario could be that at the peak of an acute inflammation, when inflammatory monocytes have concluded their role, endogenous corticoids and IL-10, involved in the resolution phase, increase the number of TRAIL-Rs on monocytes and facilitate their apoptotic death and final disposal. If this is the case, it would be of interest to investigate TRAIL-R expression in monocytes/macrophages under conditions of chronic non-resolving inflammation.

The other interesting result of this study was the finding that, in both mice and humans, tissue resident macrophages do not usually express TRAIL-Rs (with the exception of murine splenocytes), while macrophages infiltrating the tumor micro-environment are clearly TRAIL-R positive. This divergent expression could be explained with the different origin of resident peripheral macrophages (mainly embryonic precursors derived from the yolk sac) and of TAM (circulating monocytes, which express TRAIL-R1-2) [49]. Furthermore, the tumor micro-environment is usually rich in IL-10, which is the major regulator of TRAIL-Rs among the factors tested in this study.

Another myeloid cell type associated to cancer is the heterogeneous population of MDSC. Condamine et al reported that MDSC cells from tumor-bearing mice are susceptible to TRAIL-induced apoptosis and that under conditions of endoplasmic reticulum (ER) stress the DR5 receptor is up-regulated [50]. In our study, MDSC from spleens of tumor-bearing mice expressed low levels of DR5 and to a much lower extent compared to macrophages. It may possible that different isolating techniques and distinct anti-DR5 antibodies may account for this discrepancy. In the tumor micro-environment, Wilson et al. reported that endothelial cells in cancer-associated vessels express DR5 and that TRAIL may function as a tumor vascular disruption agent [51]. In our fibrosarcoma model, we only sporadically observed receptor positivity co-localized with CD31 staining; however, a marked effect was found on the vessel network after *in vivo* TRAIL treatment, although it cannot be excluded that this is an indirect effect caused by the reduced macrophages.

The finding that TAM have functional TRAIL-Rs bears interest for therapeutic implications. In the last few years it has become increasingly clear that targeting of tumor macrophages is a promising approach to limit tumor progression. The most studied strategies have used inhibitors to the receptor kinase specific for mononuclear phagocytes (M-CSF-R). Several different inhibitors are now available; including monoclonal antibodies and small compounds, and their effects in experimental mouse tumor models is highly promising. Another approach is to limit the recruitment of circulating monocytes at tumor sites by interfering with their response to chemoattractants [13, 52], or to re-activate macrophage cytotoxic potential with agonist anti-CD40 antibodies, TLR agonists or low dose irradiation [53, 54].

As a proof of principle that TRAIL-Rs on TAM constitute an interesting molecular target to induce their apoptosis, we treated tumor-bearing mice with recombinant TRAIL and observed a significant decrease of TAM and of circulating monocytes; even if tumor cells were completely resistant to TRAIL-induced apoptosis, a significant slow down of primary tumor growth was noted, as well as a reduction in the number of spontaneous lung metastasis. These results, therefore, constitute the rationale to consider TRAIL therapy as an approach to target TAM, even in contexts where tumor cells have acquired resistance to TRAIL although this remains to be demonstrated in more relevant clinical settings.

In general, TAM have a M2-like phenotype but their heterogeneity is emerging in the last years and different patterns can be found in different tumor types as well as along tumor progression [4, 55]. In our fibrosarcoma model, the receptor DR5 was predominantly expressed by CD206^+^ Tie2^+^ TAM rather than by the fewer MHC II^high^ TAM; thus DR5+ TAM displayed a typical phenotype of M2-polarized macrophages [7, 56]. This was in part consistent with the results of *in vitro* differentiated human macrophages where TRAIL-R2 was indeed higher in M2 than in M1 macrophages.

There is considerable interest in the potential role of TRAIL in cancer therapy [24, 26, 27, 57–59]. Although in the past 15 years the clinical results have been quite disappointing, as many tumors showed intrinsic or acquired resistance to TRAIL treatment, new recombinant forms of TRAIL and agonistic antibodies are under clinical evaluation for a number of different tumors [26, 57, 58]. To counteract TRAIL-resistance in tumor cells, two strategies have been implemented: combining TRAIL therapy with other anti-tumor therapies [60] or with natural compounds that increase cell susceptibility [61]. For instance, Apigenin, Quercitin and Palmitate have been shown to increase the expression of functional TRAIL-Rs on the surface of tumor cells and to restore TRAIL-induced apoptosis [32, 62, 63]. We found in this study that these natural compounds, as well as trabectedin, up-regulated TRAIL-Rs on monocytes, and in some cases were able to induce co-localization of TRAIL-Rs into lipid rafts and trigger apoptotic death.

In conclusion, the expression of functional TRAIL-Rs on cells of the mononuclear phagocyte system and their susceptibility to apoptosis indicate that TRAIL-based therapies could be an alternative approach to target macrophages in tumors.

## MATERIALS AND METHODS

### Drugs and stimuli

For *in vitro* experiments Recombinant Super Killer Trail (Human: Enzo Lifesciences; Mouse: Adipogene) was dissolved in PBS−/− 1%BSA, kept at −80°C and used 300 ng/ml.

For *in vivo* experiments Trail Recombinant *Killer*TRAIL (Alexis) was resuspended in PBS−/− and used 10 mg/kg i.p every 3 days for 2 weeks.

Glucocorticoids (MP Biomedicals) were used 100uM; hIL-10, hIL-1, hIL-2, hIL-4, hIL-6, hTNFα, hINFγ, hTGFβ (Peprotech) 25 ng/ml; LPS (Alexis) 100 ng/ml; Pam3Cys (Vinci Biochem) 2 ug/ml according to the manufacture's instructions.

Apigenin, Quercitin (Indena) and trabectedin (Pharmamar) were resuspended in DMSO and used 40uM, 400uM and 10 nM respectively. Palmitate (SIGMA), Indole-3-Carbinole (I3C) resuspended in DMSO and used 800uM and 100uM respectively.

### *In vivo* experiment

The transplantable MNMCA-1 mouse fibrosarcoma were inoculated intramuscularly (5×10^4^ cells) in C57/BL/6J mice (Charles River - Calco, Como Italy) and tumor growth was observed over 3 weeks. All mice were used between 6-10 week of age.

Mice, Tumors, and Primary mouse Cells were used in compliance with national (4D.L.N.116, G.U., suppl. 40, 18-2-1992) and international law and policies (EEC Council Directive 86/609, OJ L 358, 1, 12-12-1987; NIH Guide for the Care and Use of Laboratory Animals, US National Research Council, 1996). This investigation was approved by the Animal Care and Use Committee of the Humanitas Clinical and Research Center.

### Cell isolation, cell culture and tissue samples

Human monocytes, neutrophils, and T-lymphocytes from blood of healthy donors were purified through density gradients, as described in Allavena [64], from buffy-coats. Human macrophages were differentiated *in vitro* from blood monocytes cultured with 25 ng/ml M-CSF for 5 days and then polarized with LPS (100 ng/ml) + IFN-γ (500 U/ml) for M1 macrophages and with IL-4 (20 ng/ml) to obtain M2 ones. Human lymphocytes were antivated *in vitro* with Ionomycin (500 ng/ml) and PMA (50 ng/ml).

Mouse blood cells were collected from the eye vein of anesthetized mice and splenocytes from disaggregated spleen and filtered through Falcon strainers. Mouse tumors were cut into small pieces, disaggregated with collagenase (0.5 mg/ml), and filtered through strainers.

Human tumor tissues and adjacent un-diseased tissues were obtained from patients who underwent surgical treatment in our Institute. Murine normal tissues (spleen, skin, liver, lungs) were obtained from 6 weeks old C57/BL/6J mice from the animal house of our institute. Samples were stained with specific antibodies.

### Antibodies

To study the expression of death receptors in human leucocytes, cells were resuspended in PBS−/− 1X 1% FBS and stained with anti-human TRAIL-R1, TRAIL-R2 (Enzo Lifesciences), anti-human TRAIL R3 (R&D Systems) and anti-human Fas (Millipore) used according to the manufacture's instructions. To block their activity were used recombinant human Trail R1/R2 Fc chimera (R&D Systems), anti-human TRAIL R3 (R&D Systems) and anti-human Fas (Millipore).

To analyze cell phenotype in mouse blood, spleen and tumor were used PerCp-Rat CD45 (30F11), FITC Hamster CD3 (145-2C11), PE Rat Ly6C and Ly6C (RB6-8C5), FITC Rat Ly6C (AL-21), PE-Cy7 Rat Ly-6G (1A8), FITC Rat I-A/I-E (2G9) as well as relative control antibody, streptavidin dye conjugated (APC) (BD Biosciences);Anti-mouse PE CD115 (AFS98), Alexa Fluor 647 CD19 (1D3), PE Rat CD202b/Tie2 (Tec4) (eBioscience); Anti-mouse PE F4/80 (A3-1), FITC Rat CD206 (MR5D3) (AbD Serotec); Biotin-RatCD253(DR5) (N2B2) (Miltenyi Biotec) and finally, Pacific Blue-Rat CD11b (M1/70) (Biolegend). Labeled cells were fixed in PBS−/− 1X 1% formalin. After staining procedures, acquisition was performed by FACS CantoII instrument (BD Biosciences) and analyzed by FACS Diva and FlowJo software version 6.1.1 (BD Biosciences)

To perform immunofluorescence on human tissues were used anti-TRAIL-R1, TRAIL-R2 (Enzo Lifesciences), anti- TRAIL R3 (R&D Systems), rabbit CD68 (PGM1), rabbit myeloperoxidase, and rabbit CD3 purchased by DAKO; Lipid rafts are detected using Vibrant Lipid Raft Labeling Kits (molecular brobe) used according to the manufacture's instructions.

For mouse tissues were used goat TRAIL R2 (DR5), goat CD31 (R&D Systems), rat Ly-6G (1A8) (BD Pharmingen) and rat F4/80 (Serotec).

### Apoptosis and cell death analysis

Apoptosis was evaluated through Annexin V/PI staining using the specific detection kit (Immunostep) used according to the manufacture's instructions.

To evaluate caspase8 activation were used anti-human cleaved-caspase8 (18C8) anti-mouse cleaved-caspase8 Xp Rabbit Mab (D5B2) (Cell signaling) and Fixation/Permeabiliation solution kit (BD Biosciences) according to the manufacture's instructions. Labeled cells were fixed in PBS−/− 1X 1% formalin.

### Statistical analysis

Statistical analysis was performed using a paired Student's t-test. P-values of less than 0.05 were considered significant

## SUPPLEMENTARY FIGURES


