# Supplementary material for: Functional TRAIL receptors in monocytes and tumor-associated macrophages: A possible targeting pathway in the tumor microenvironment
Source: Oncotarget. 2016 May 13;7(27):41662–76. doi: 10.18632/oncotarget.9340 (PMC5173086; doi:10.18632/oncotarget.9340)
Supplement: Supplementary file 1 [file oncotarget-07-41662-s001.pdf]

SUPPLEMENTARY FIGURES

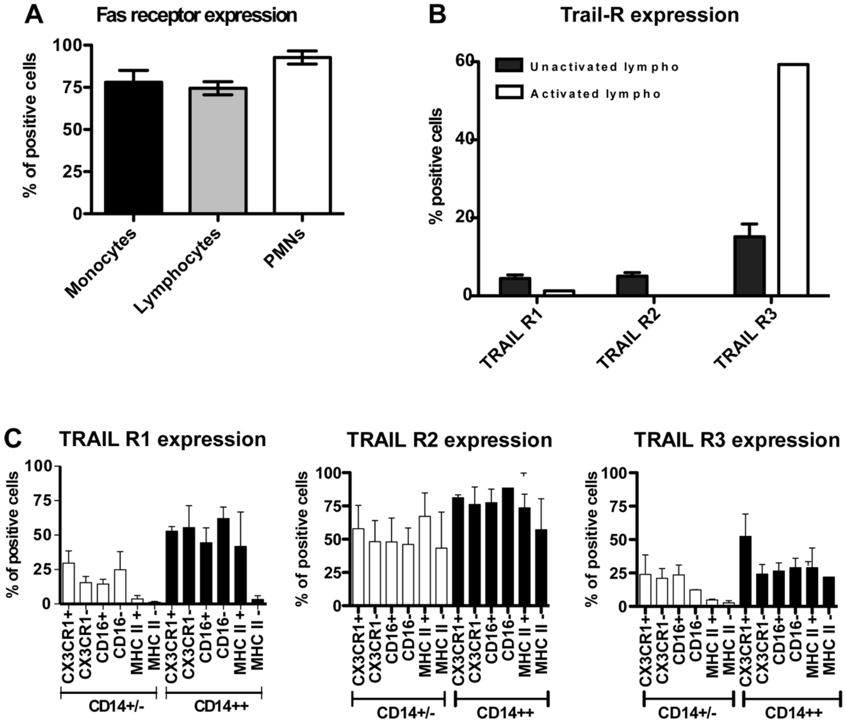

Supplementary Figure S1: Expression of death receptors in leukocyte subsets. A. Flow cytometry analysis of Fas receptor in leukocyte subsets; results are shown as % of positive cells (mean±SE of 4 experiments). **B.** Flow cytometry analysis of TRAIL receptors in resting and PMA-activated lymphocytes. **C.** Flow cytometry analysis of TRAIL receptors in monocytes subsets according to the expression of CD14, CD16, MHC II and CX3CR1; results are shown as % of positive cells (mean ± SE of 3 experiments).

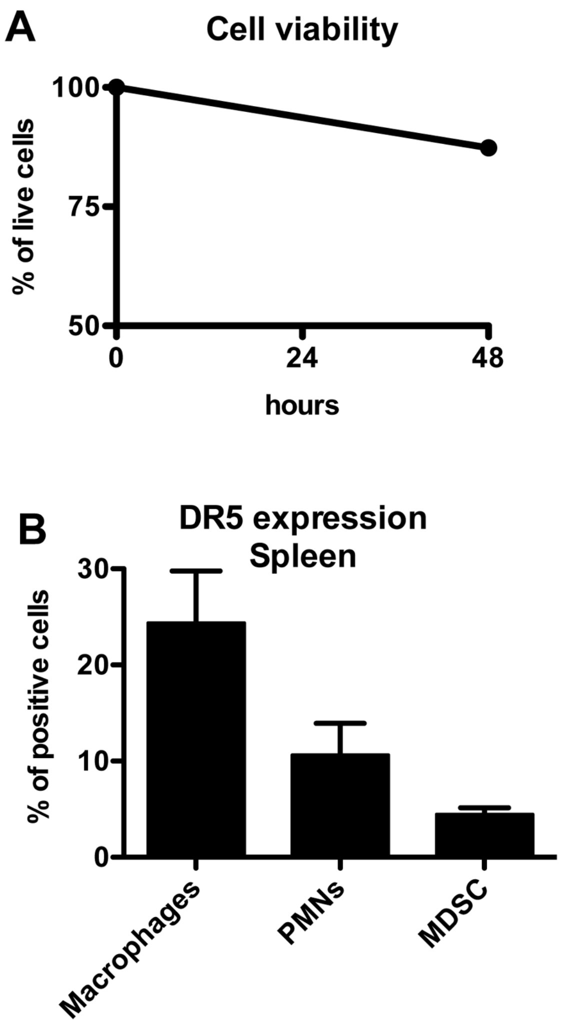

Supplementary Figure S2: Murine monocytes/macrophages express DR5 and are susceptible to TRAIL-induced apoptosis. A. Cell viability analyzed by flow cytometry of murine bone marrow-derived monocytes after TRAIL treatment (300 ng/ml) *in vitro*; results are shown as % of live cells (mean ± SE of 4 experiments). **B.** Flow cytometry analysis of DR5 in murine spleen macrophages, granulocytes (PMNs) and MDSC; results are shown as % of positive cells (mean ± SE of 6 experiments).

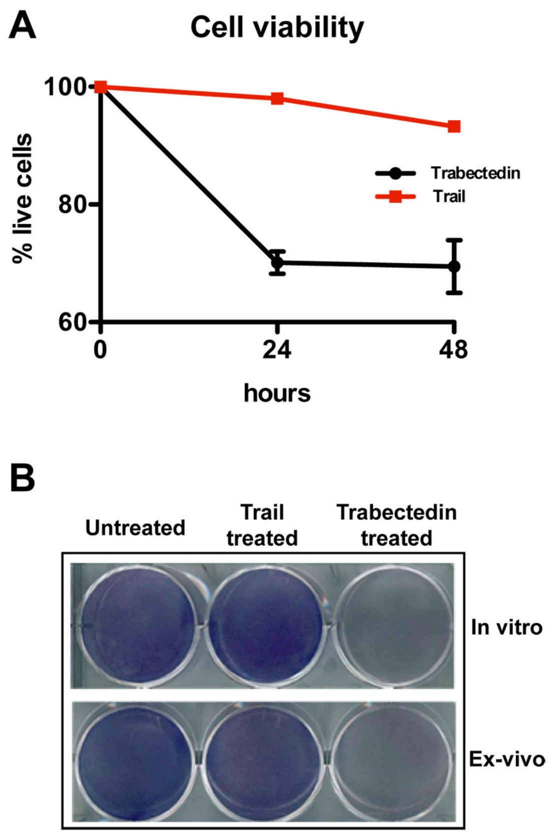

Supplementary Figure S3: Resistance to TRAIL-induced apoptosis of fibrosarcoma cells. A. Cell viability assay evaluated with Annexin/PI staining in flow cytometry of MN-MCA1 fibrosarcoma cells. Tumor cells are resistant to TRAIL treatment (300 ng/ml) *in vitro* but are susceptible to trabectedin (10 nM) (mean ± SE of 2 experiments). **B.** Explanted tumors after *in vivo* growth were cultured *in vitro* in the presence of TRAIL (300 ng/ml) or trabectedin (10 nM); culture plates were stained after 48 hrs.
